# Improvements in Fast Mass Microscopy for Large-Area Samples

**DOI:** 10.1021/acs.analchem.4c03480

**Published:** 2024-10-28

**Authors:** Edith Sandström, Pascal Huysmans, Frans Giskes, Paul Laeven, Sebastiaan Van Nuffel, Ron M. A. Heeren, Ian G. M. Anthony

**Affiliations:** † The Maastricht MultiModal Molecular Imaging Institute (M4i), Division of Imaging Mass Spectrometry, Maastricht University, Maastricht 6229 ER, The Netherlands; ‡ Instrument Development, Engineering & Evaluation (IDEE), Maastricht University, Maastricht 6229 ER, The Netherlands

## Abstract

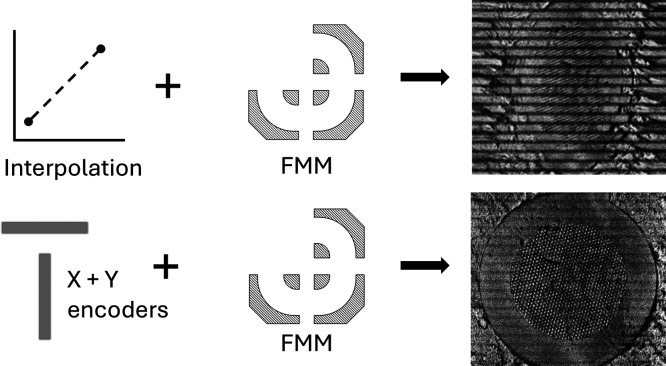

Mass spectrometry imaging (MSI) is a technique that analyzes the chemical information and spatial distribution of surface analytes. Most MSI studies are conducted in microprobe mode, in which a mass spectrum is collected for each pixel to create a mass image. Thus, the spatial resolution, sample imaging area, and imaging speed are linked. In this mode, halving the pixel size quadruples the analytical time, which presents a practical limit on the high spatial resolution MSI throughput. Fast mass microscopy (FMM) is, in contrast, a microscope-mode MSI technique that decouples spatial resolution and imaging speed. FMM circumvents the linear-quadratic relationship of pixel size and analytical time, which enables increased imaging size area and the analytical speed achievable. In this study, we implement instrument modifications to the FMM system, including the addition of linear encoders that enable roughly 8.5× faster imaging than was previously achieved, allowing a 42.5 × 26 mm^2^ sample area to be imaged at a 1 μm pixel size in <4.5 min. Linear encoders also enable the alignment of multipass images that increase image homogeneity and signal intensity. The applicability of FMM to large area samples has made it important to define the tolerance to height variations of the technique, which was determined to be at least 218 ± 0.03 (*n* = 3) μm.

Mass spectrometry imaging (MSI) is an analytical technique that obtains chemical information along with its spatial distribution.^1,2^ The ability of MSI to simultaneously image thousands of molecules in a single experiment makes it a powerful tool for fields varying from biomedical^3−5^ and forensic^6,7^ to material^8^ and heritage^9−11^ sciences. The most common mode of MSI is microprobe mode, wherein a laser or ion beam is scanned serially over the sample collecting a mass spectrum per pixel.^12^ Halving the pixel size in microprobe mode thus requires quadrupling the number of pixels and thus the analysis time. This linear-quadratic relationship limits the size of the sample area that is practical to analyze by MSI in microprobe mode at high spatial resolutions.^13,14^

A strategy to circumvent this linear-quadratic relationship is microscope-mode MSI, also known as mass microscopy,^15^ in which a defocused ion beam generates ions from the sample surface under high vacuum conditions. The relative spatial locations of the ions are preserved using stigmatic ion optics during time-of-flight (TOF) mass analysis.^13,15^ The mass and spatial information were then collected simultaneously by a pixelated TOF detector. An advantage of microscope-mode MSI is that the pixel size of images generated is independent of spatial resolving power and beam spot size.^16−20^ As the signal intensity still follows the square rule, mass microscopy is not unlimited in the spatial detail able to be provided.

Fast mass microscopy (FMM) is a microscope-mode MSI technique that uses a fast-moving sample stage and a fast pixelated detector, such as a TPX3CAM, to allow the investigation of larger areas at short time scales. Despite its name, FMM is an MSI technique, which differs from microscopy techniques by the collection of mass spectral information. In a previous study, FMM was used to image a 42 × 23.5 mm^2^ area in 33.5 min at a pixel size of 0.9 μm.^21^ This speed is orders of magnitude faster than microprobe mode MSI. Although fast, the previously reported FMM results were still limited in speed, because they made use of estimating the sample stage location via interpolation to position the ion images. Estimates of velocities become less accurate and precise at higher speeds. Furthermore, due to variations in motor acceleration and timing, the estimation was also not reproducible at the 1 μm scale between replicate measurements.

Herein, we remove the need for interpolation using linear encoders that provide the exact positions of the sample stage for each TOF cycle. We use the encoder-provided coordinates to image a 42.5 × 26 mm^2^ area in <4.5 min at a pixel size of 1 μm, which corresponds to approximately an 8-fold speed increase compared to the previously reported study. Using the precise positioning of the encoders, we demonstrate that multiple fast passes of a large area can be taken quickly and improve the quality of the image reconstruction. As a result, image homogeneity is improved, as measured by standard deviation, and a dynamic-time mode of imaging is enabled. Additionally, we investigate the limits of topographical differences of the FMM instrument, as these are relevant for reliable imaging of large-area samples.

## Experimental Methods

### Instrumentation

The BioTRIFT system used includes a C_60_ ion beam (IOGC60-20S, Ionoptika, Chandler’s Ford, UK) and a Timepix3 ASIC-based camera^22^ (TPX3CAM, Amsterdam Scientific Instruments, Amsterdam, NL) and is described in detail elsewhere.^21^ For the TRIFT system, in principle, mass resolution is 12,000 *m*/Δ*m*_50%_ at *m*/*z* 28.^23^ However, in practice with the Timepix3 setup and speed-based optimizations, mass resolutions are between 10 and 600 *m*/Δ*m*_50%_ at *m*/*z* 400, depending on primary ion pulse duration (between 500 and ∼12 ns, respectively). All primary ion pulses used for this work were between 150 and 500 ns. Two linear encoders (LS 477, Heidenhain, Traunreut, DE) connected to a digitization card (NI6612, National Instruments, Austin, TX, USA) were added to the BioTRIFT stage directly under the existing encoders, allowing both normal instrument operation and recording of stage coordinates (Figure S1). The addition of the new encoders allowed for instrument stage control to continue to function normally and without any hardware or software modifications. A NI6612 digitization card was used in conjunction with the new encoders to save the *X* and *Y* coordinates of the stage for each TOF cycle. Software performing this synchronized collection was written in LabVIEW 2020 32-bit (National Instruments, Austin, TX, USA) as a custom program, and an overview of the workflow is included in the Supporting Information (Figure S2). These coordinate files were combined with the respective TPX3CAM.tpx3 file in postprocessing to produce the mass images. The postprocessing workflow is described previously^21^ but has been modified to either use the coordinates from the encoder files or to use the interpolated stage position (e.g., if no encoder files were collected). The primary TOF trigger was split using a Standford Research Systems DG535 delay generator, and the resulting split signals were supplied to the C_60_ ion gun pulser, the TPX3CAM detector TDC input, and the NI6612 digitization card.

A FIB-SEM (Scios 2 DualBeam system, Thermo Fisher Scientific, Waltham, MA, USA) was used to measure the height at points on each stacked grid relative to the ITO slide. The SEM micrograph of the stack of grids can be seen in Figure S3.

### MSI

The C_60_ ion beam aperture was set to 1 mm, resulting in a field of view of approximately 320 μm in diameter and a continuous ion beam current of 0.5–0.8 nA at a source temperature of 410 °C. The largest contrast diaphragm aperture of the BioTRIFT was used for all images.

### Materials

A hexagonal 400 mesh thin bar Cu grid was imaged to compare images with and without the encoder data. Hexagonal 200 mesh Cu grids were used for the stack of the grids. A hexagonal 460 mesh Cu grid and a bar 400 mesh Ni grid were added to the composed surface sample. All TEM grids were obtained from Agar Scientific, UK. Indium tin oxide (ITO)-coated glass slides (Delta Technologies, Loveland, CO, USA) were used for all samples. Gd(III)Cl_3_·6H_2_O, Ho(III)Cl_3_·6H_2_O, and red phosphorus were purchased from Merck, Germany. The lemon and table salt (NaCl) were purchased locally.

### Sample Preparation

For comparison of image construction with and without encoder-supplied coordinates, an ITO-coated glass slide was prepared with a TEM grid secured with ink from a highlighter marker.^24^ To determine the focal depth tolerance, on a separate ITO slide, five TEM grids were stacked on top of each other. Each grid was adhered with a highlighter marker before the next was placed on top off-center to create a “staircase” shape approximately 200 μm in height measured from the surface of the ITO-coated slide. To add additional stack height, the top grid was bent slightly before placement on the stack of grid to create a “ramp” in the *z*-direction of approximately 200–500 μm in height measured from the surface of the ITO-coated slide.

For the “composed surface” data set, a lemon purchased from a local supermarket was cut into wedges. The wedges were loosely wrapped in aluminum foil and placed in a container of dry ice for 1 h to freeze. A Microm HM535 cryomicrotome (Microm International) at a temperature of −20 °C was used to produce 50 μm thick sections. The sample was then thaw-mounted onto a clean ITO-coated glass slide and immediately placed into a vacuum desiccator to dry for 30 min before storage at −80 °C.

On the day of the measurement, a saturated aqueous solution of NaCl was prepared, and the operator’s thumb was immersed in the saturated NaCl solution before being pressed next to the lemon to produce a fingerprint. Two TEM grids were placed on the slide and then adhered by dabbing them with a highlighter marker. Approximately 100 μL of an aqueous GdCl_3_ solution (10 mg mL^–1^) and an aqueous HoCl_3_ solution (10 mg mL^–1^) were pipetted onto the slide. The slide was then allowed to dry for 1 h at room temperature and pressure, and finally, ∼70 mg of red phosphorus was sprinkled across the slide. In general, mass microscopy is difficult or impossible to perform with nonconducting surfaces because surface charging causes distortion to the ion images. Therefore, the slide was sputter coated with a 1.1 nm Au layer (SC7640, originally Polaron Ltd., now Quorum Technologies, Laughton, UK) before the measurement. The intention behind this composed surface sample was to produce a large area “test” sample with distinct mass images and fine spatial features that were resolved in the mass domain and partially resolved in the spatial domain.

### Measurement

All measurements presented used a *m*/*z* range of 0.5–200 Da. The singular TEM grid was imaged with settings of 350 shots per area, a C_60_ pulse time of 300 ns, and a row overlap of 90%. The measurement of the stack of grid settings was 10,000 shots per area, C_60_ pulse time of 300 ns, and row overlap of 75%. The composed sample was first imaged with 300 shots per area, a C_60_ pulse of 500 ns, and a row overlap of 30%. The investigated area was 42.5 × 26 mm^2^ measured over 264.6 s. Six subsequent images of the composed sample surface were imaged with varying settings of pulse times of 150–500 ns, row-overlaps of 30–40%, and 300 shots per area. Primary ion doses were between 3.72 × 10^8^ and 1.3 × 10^9^ ions cm^–2^ for the seven images. No active charge compensation was employed during any of the measurements; the Au coating was noted to improve performance on nonconducting surfaces and functioned to assist in passively dispersing charge.

## Results and Discussion

### Advantages of Linear Encoder Coordinates

Linear encoders were installed beneath the original stage encoders of the fast mass microscope (Figure S1). The use of encoders is common in imaging and manufacturing positioning equipment because knowledge of the precise position is of importance.^25^ Recently, their introduction with the microgrid technology (Bruker, Billerica, MA, USA) facilitates high quality <5 μm spatial resolution MALDI-MSI analysis by enabling more accurate laser beam positioning.

**Figure 1:**
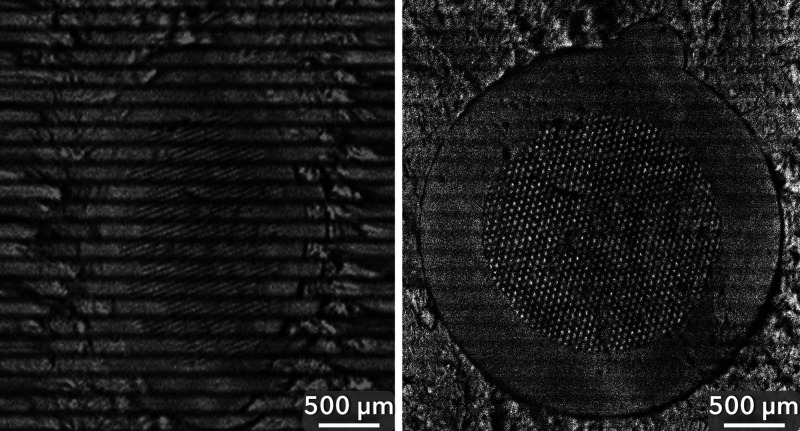
FMM total ion count (TIC) image of a TEM grid constructed by the previous method of coordinate estimation by interpolation (left) and the use of the exact encoder coordinates (right)**.** The same TPX3CAM data was used for both images.

The interpolation method of FMM image construction assumes constant stage and sample velocity.^21^ By knowing the length, start, and end time of each row imaged, the position of the individual ion images can be estimated by linear interpolation. It was observed that image artifacts and errors increased with higher stage speeds as well as shorter row lengths (Figure 1). Under these conditions, the assumption of constant stage and sample velocity does not hold, since relatively more time is spent accelerating/decelerating and traveling between rows. With the encoders, each TOF cycle has a corresponding set of coordinates that enable accurate positioning of ions at any achievable stage velocities—e.g., a maximum of 3.2 cm s^–1^ was recorded on the BioTRIFT instrument, but higher velocities should be possible.

The effect of the encoder coordinates on the total ion image was tested on a 4 × 4 mm^2^ area containing a TEM grid, imaged in 38 s at a speed of 0.42 mm^2^ s^–1^ (420,000 pixel s^–1^ at 1 μm pixel size). The same TPX3CAM data was used for both images in Figure 1. The use of the interpolated coordinates (Figure 1, left) is clearly inferior and leads to row mismatch, blur, and image distortion when compared to the use of exact encoder coordinates (Figure 1, right). At lower speeds or longer rows, the image construction using interpolation works sufficiently. The poor quality in Figure 1, left is due to the nonlinear nature of the scanning acceleration and deceleration.

The horizontal bands seen in both constructed images in Figure 1 can be reduced by optimizing the row overlap or by the use of multiple imaging scans. However, at high speeds, alignment of multiple imaging scans is only feasible by the incorporation of the encoder coordinates, which makes the imaging measurement reproducible and more accurate.

### Measurement of FMM Focal Depth

In microprobe MSI, variation of surface height of even a few micrometers may cause changes in beam focus leading to loss of sensitivity.^23,26,27^ This problem is severe enough that multiple microprobe systems incorporate autofocusing systems to enable imaging of uneven surfaces, often by incorporating a third axis into their imaging stages to enable compensating for surface height variation.^28−31^ In a previous work, we noted that the use of an already defocused beam, such as that used in mass microscopy, reduces the negative effects of beam defocusing due to topographical differences across a sample.^21^

This tolerance to topographical height differences is of particular interest to large spatial area imaging applications because larger areas tend to have more surface height variations than local areas. In the authors’ experience, MSI-prepared biological tissue samples are sectioned at thicknesses at or below 50 μm. Thus, a large-area imaging technique for biological tissues should ideally be robust at this height. The topographical tolerance of the FMM system was investigated on TEM grids stacked on top of each other with the top grid bent upward, creating a surface with a height variation of at least 484 μm.

**Figure 2:**
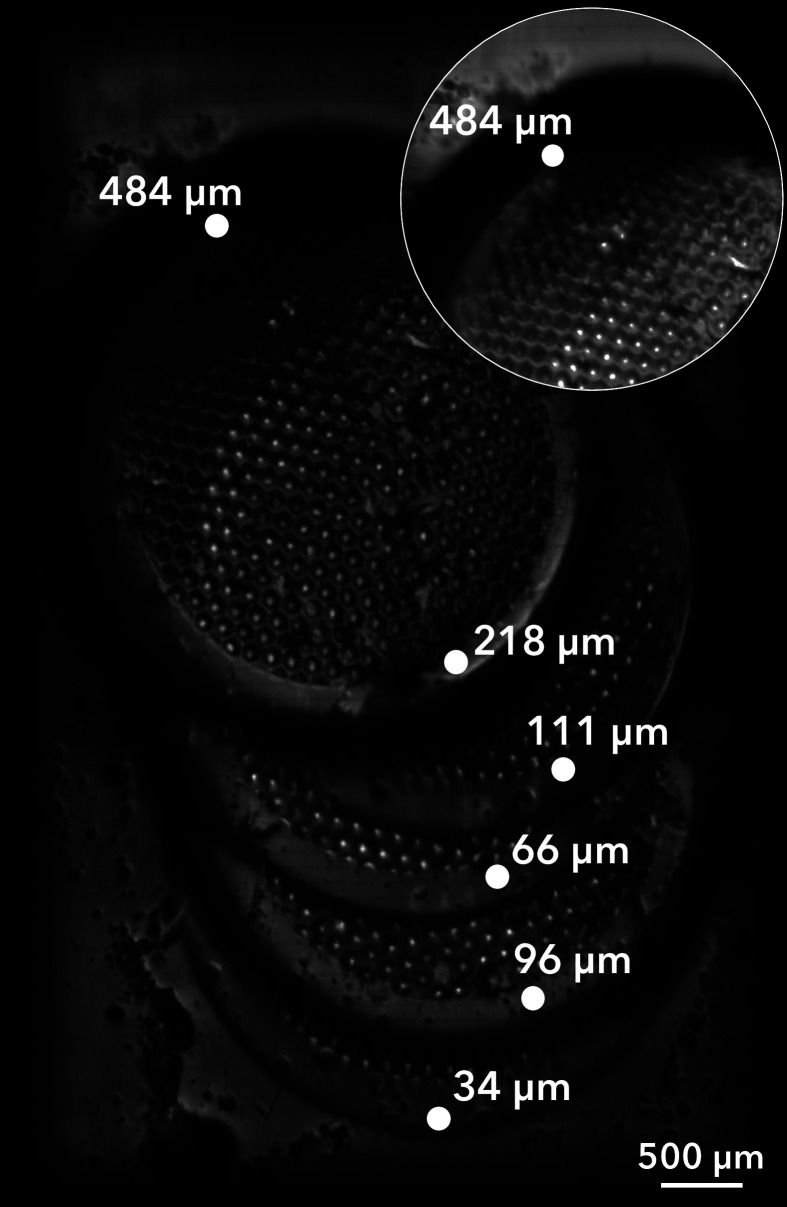
FMM TIC image of five TEM grids stacked on top of each other, showing the height tolerance of the method to be at least 218 μm. A digitally brightened zoomed-in view of the highest point of the bent grid is included in the top-right corner. The height of points on each TEM grid was determined by SEM analysis, with an absolute uncertainty range of 0.012–0.068 μm (*n* = 3) for all measurements.

Figure 2 shows an FMM TIC image of the stack of the TEM grids. Spots on the stack of grids were measured for their surface height using SEM. The total ion intensity and image sharpness appear no different between 0 and 218 μm of surface height. However, a decrease in signal intensity and an increase in image blur can be observed at 484 μm, highlighted in a brightened inset in Figure 2. For this image, the BioTRIFT ion optics were optimized for features at a height of 0 μm (i.e., the ITO slide), which means that the total effective focal depth and high sensitivity region of the FMM system may extend below the “0 μm” point.

The mass spectra obtained from the top and bottom grids in the stack of grids have the same overall appearance (Figure S4). The bent shape of the top grid seemed to have no large noticeable effects except for the expected gradual loss of signal going up the bend. Thus, the main effect of the height difference was the signal intensity, with the bottom grid spectrum showing four times higher relative intensity compared to the mass spectrum collected from the top grid. There was also a small peak shift (∼0.05 Da at *m*/*z* 23) toward larger *m*/*z* values of the mass spectrum from the bottom grid compared to the top grid. Correcting such peak shifts has been reported to increase the mass resolution and sensitivity of MSI experiments.^23^ However, the poor mass resolution of the current experimental setup would not benefit substantially from such a correction.

Although the sensitivity and image focus were not observably altered between 0 and 218 μm, the location of the primary ion beam (and thus of the ejected ion images) changes depending on surface height. This surface-height shift may be easily corrected by adjusting the primary ion beam deflection optics to keep the beam centered. However, if these adjustments are not performed, then the edges of the ion images will drift outside the field of view of the detector, leading to lower signal intensity in these areas. Figure S5 demonstrates this effect when the stack of five TEM grids shown in Figure 2 is imaged, and no beam position adjustments are performed.

### Improved Speed of Imaging of a Large Area

**Figure 3:**
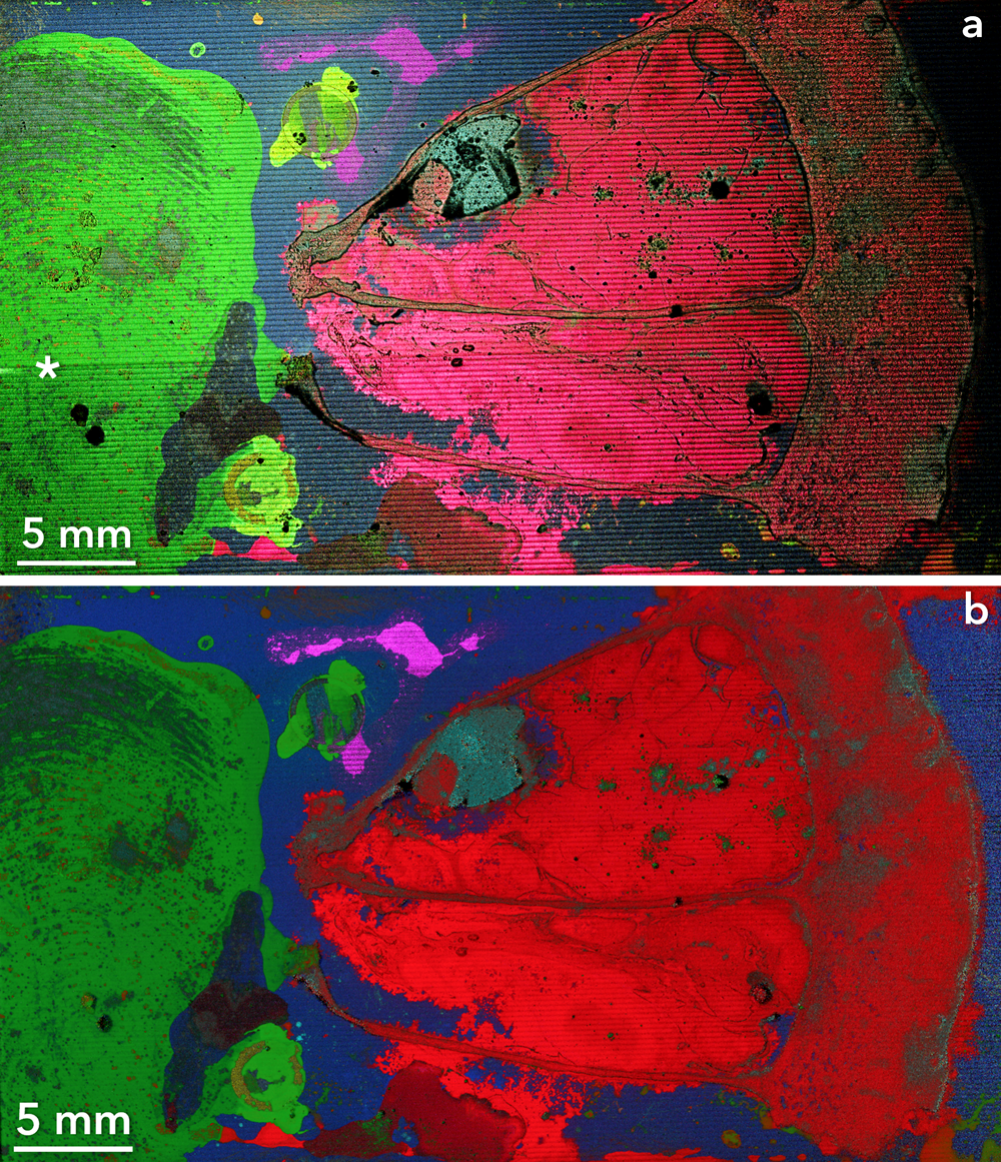
Mass colored FMM images of a 42.5 × 26 mm^2^ area of the “composed surface” including Na^+^ (green, present primarily in the fingerprint), HoO^+^ (magenta, present above and around the top TEM grid), In^+^ (blue, present as a background), Cu^+^ (yellow, present in the lower TEM grid), K^+^ (red, in the lemon section), and *m*/*z* 57, an organic fragment (cyan, present in the lemon section’s seed). (a) Single-pass ion image that was acquired at 4.18 mm^2^ s^–1^ in under 4.5 min. The asterisk in (a) indicates the horizontal row below which the image has been digitally brightened to match the top of the image due to detector instability. (b) Multipass ion image composed of seven measurements all of the same area as the single-pass image. The total imaging time for all seven passes of the image in (b) was 32.75 min, a rate of 0.562 mm^2^ s^–1^.

A composed surface containing a 50 μm thick sectioned lemon slice combined with other elements with unique chemical and spatial features was prepared to test the practicality of the improved speed enabled by the encoders and the ability of FMM to image large-area biological tissues. An area of 42.5 × 26 mm^2^ was imaged with a measurement time of 264.6 s which is a rate of 4.18 mm^2^ s^–1^ (Figure 3a). This imaging rate is approximately 8.5 times faster than the previously reported 0.49 mm^2^ s^–1^.^21^ At a pixel size of 1 μm, this image is >1.1 billion pixels, and the pixel acquisition rate is 4.176 million pixels s^–1^. For reference, currently, fast microprobe-mode MSI is generally restricted to imaging below 1000 pixels s^–1^.^32−34^ Even assuming a single TOF cycle per mass spectrum, for a hypothetical instrument if each TOF cycle requires 100 μs, microprobe mode is mathematically incapable of imaging faster than 10,000 pixels s^–1^. Although 4.176 million pixels s^–1^ was demonstrated here, it is likely that improvements in ion image field of view and instrument optimization could increase FMM to be well over 10 million pixels s^–1^. Optimizable parameters that may improve FMM speed are increases in motor speed, higher voltage electronics to enable shorter TOF cycles and corresponding improvements in detector technology such as the Timepix4.^35^

The impact of instrumental variations and image artifacts, such as the row pattern observed in Figure 1 and in zoom-ins of Figure 3 (Figure S6), can be decreased by the addition of multiple passes of the same area. These multipass acquisitions are feasible because of the exact coordinates provided by the encoders. Seven measurements of the composed surface sample were collected in 1964.9 s (32.75 min) and combined into one image (Figure 3b). Comparing this multipass image to the single run image in Figure 3a shows the clear increase in image homogeneity as well as chemical information. The image acquired in Figure 3a darkens perceptibly due to detector instability. The bottom third of Figure 3a has therefore been digitally brightened to more closely match the top of the image and show that mass information is still present despite the change in the detector response. This detector instability was present and visible in all seven individual passes acquired for Figure 3b (Figure S7 shows these individual images for selected masses and the TIC); however, the overall construction reduced the visibility of such detector instability effects on the constructed image. There is a general color shift between Figure 3a,b (most visible in the lower intensity color channels of yellow, cyan, and magenta). This color shift is attributed to the method used of digitally composing these images as the individual mass images were normalized, and the relatively higher variance of the low-intensity mass images caused a change in intensity when normalized. Individual features are also, as expected, more visible in the multipass image, especially where the total ion count is low in the individual images (Figure S6). The use of encoder coordinates serves to increase the accuracy and reproducibility between these images (Figures S7 and S8).

Multipass mode allows pausing or stopping the imaging at any time after the first pass while still resulting in complete coverage of all sample areas. This “dynamic time mode” is in contrast to the previous mode of FMM imaging, where pausing or stopping an experiment would result in an incomplete image. Furthermore, by enabling such quick scans of large areas, regions of interest (ROIs) may be able to be more easily selected and imaged at a slower rate to facilitate higher molecular information.

Additionally, the use of multipass mode allows for the combination of both higher mass resolution passes using shorter primary ion pulses and higher sensitivity passes using longer primary ion pulses (Figure S9). When added together, passes improve the number of ions observed, which improves the image contrast and chemical information. Multipass image homogeneity could also be improved beyond what is shown in this work by alternating horizontal and vertical passes rather than only using horizontal passes.

## Conclusions

The addition of exact coordinates for FMM image construction has enabled accurate image construction at higher stage velocity speeds, compared to previous work. In principle, even higher speeds may be possible, as the analytical speed is only dependent on the electrical, mechanical, and detector-based specifications of the instrument. FMM also displays a high tolerance to surface topographical variations of at least 218 μm. This was demonstrated by imaging a 50 μm thick section of lemon within a composed surface sample over a lateral distance of 42.5 mm. This sample was imaged at 4.18 mm^2^ s^–1^ and 4.176 million pixels s^–1^, which is 8.5× faster than previously reported FMM imaging and ∼4176× faster than the fastest microprobe-mode MSI instruments. Additionally, the use of exact coordinates enables multipass FMM images to be acquired as well as a “dynamic time mode” imaging. This dynamic time mode allows one to stop an imaging experiment at any point after the first pass is acquired while retaining the image of the total sample area. The application of such a mode may be helpful for time-limited studies, increasing throughput, and helping expand the MSI field further.

## Supporting Information Available

Photograph of the added encoders, screenshot of the LabView encoder readout script, SEM-micrograph of the stacked grids, FMM-TIC images of the stacked grids with region-selected mass spectra, FMM-TIC images of stack grids showing manual beam steering improvements, zoom-ins and highlighted areas of the single-pass and multipass composed surface sample mass images, small multiple images of the composed surface comparing interpolation with encoder-based construction, standard deviation images of the composed surface sample comparing interpolation with encoder-based construction, and two averaged mass spectra of the composed surface taken with 150 and 500 ns primary pulse time lengths (PDF)
Python file that includes a portion of the relevant motor and stage control code (TXT)
