# Supplementary material for: Improvements in Fast Mass Microscopy for Large-Area Samples
Source: Anal Chem. 2024 Oct 28;96(45):18037–42. doi: 10.1021/acs.analchem.4c03480 (PMC11561871; doi:10.1021/acs.analchem.4c03480)
Supplement: Supplementary file 1 — ac4c03480_si_001.pdf [file ac4c03480_si_001.pdf]

Supporting information

Improvements in fast mass microscopy for large-area samples

Edith Sandström,[a] Pascal Huysmans,[b] Frans Giskes,[a] Paul Laeven,[b] Sebastiaan Van Nuffel,[a] Ron M. A. Heeren,[a] Ian G. M. Anthony[a*]

[a]The Maastricht MultiModal Molecular Imaging Institute (M4i), Division of Imaging Mass Spectrometry, Maastricht University, Maastricht 6229 ER, The Netherlands, [b]Instrument Development, Engineering & Evaluation (IDEE), Maastricht University, Maastricht 6229 ER, The Netherlands

Table of Contents

Figure S1: S2
Figure S2: S3
Figure S3: S4
Figure S4: S5
Figure S5: S6
Figure S6: S7
Figure S7: S8
Figure S8: S9
Figure S9: S10

Figure S1:

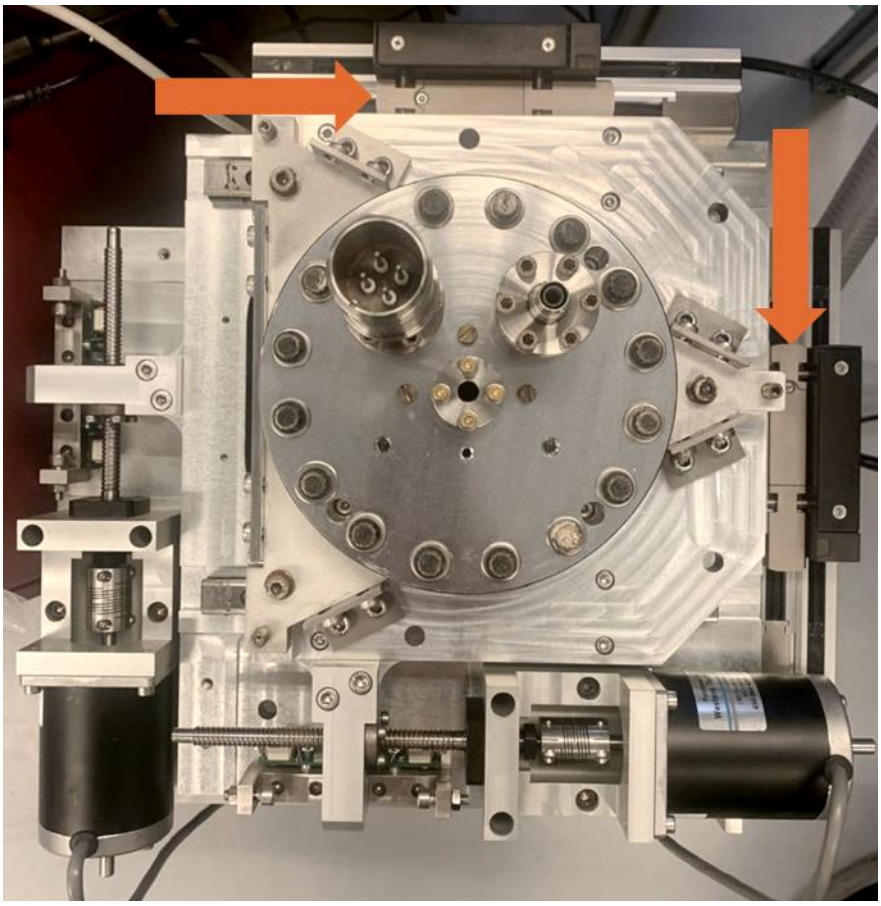

S1. Photograph of the back of the stage of the bioTRIFT instrument with the added linear encoders indicated with orange arrows. Original linear encoders are directly on top of the added encoders. This easily accomplished instrument modification was done by only using longer bolts that connect through both new and old encoders. Thus, instrument function is preserved while faster separate readout is provided. This faster separate readout is used for exact placement of individual mass images during the FMM image construction process.

Figure S2:

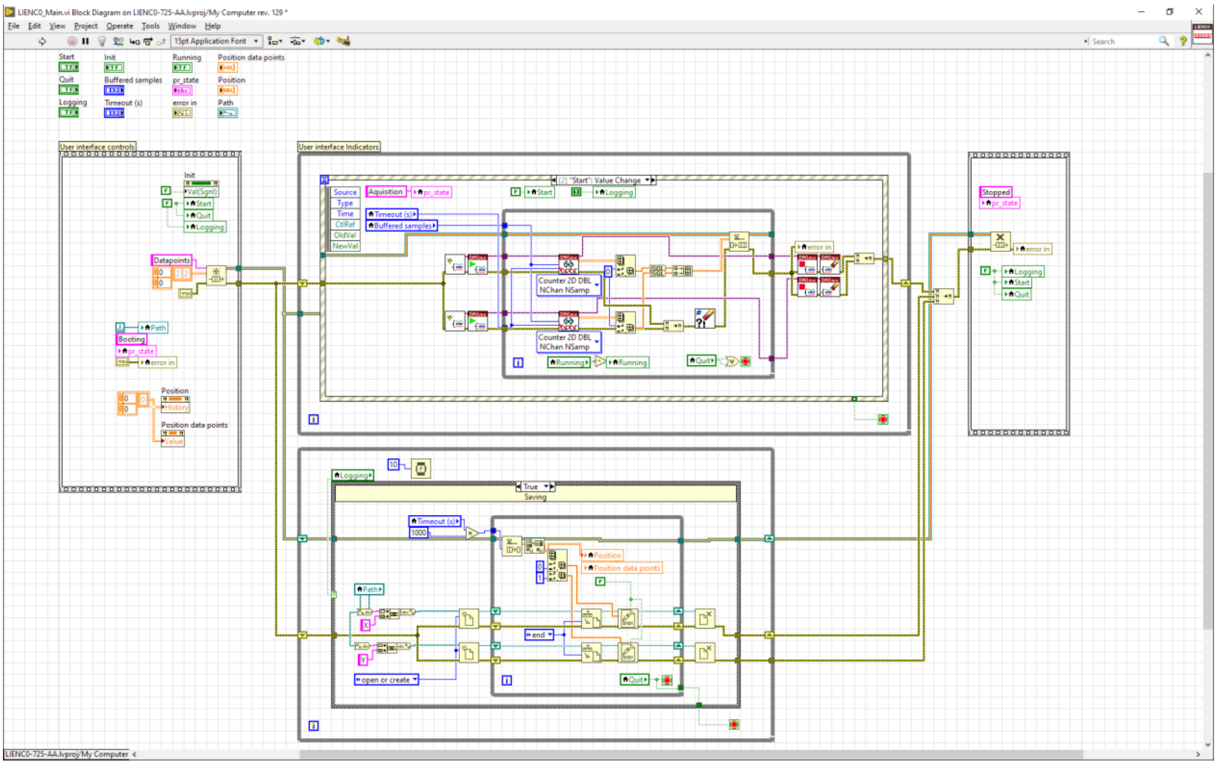

S2. Image of the LabView script used for recording the stage coordinates from the linear encoders. The script contains two main loops, one for reading the encoder signals (top) and one for saving the encoder coordinates (bottom).

Figure S3:

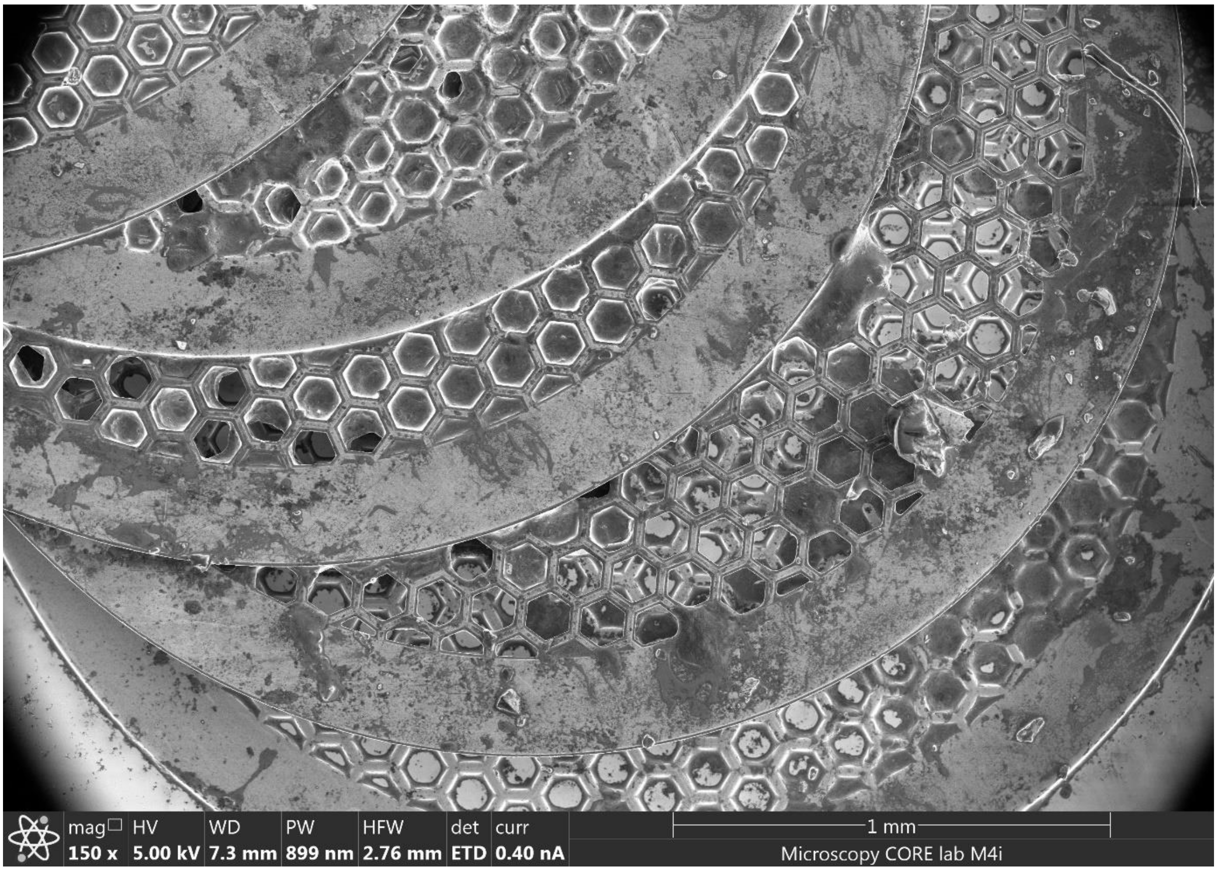

S3. SEM-ETD micrograph of the stack of grids secured to an ITO slide with ink (also shown in S4, S5, and Figure 2) point height measurements of each grid border were acquired relative to the ITO slide. Some surface imperfections and irregularities are observed in both the FMM TIC image and this SEM-ETD micrograph.

Figure S4:

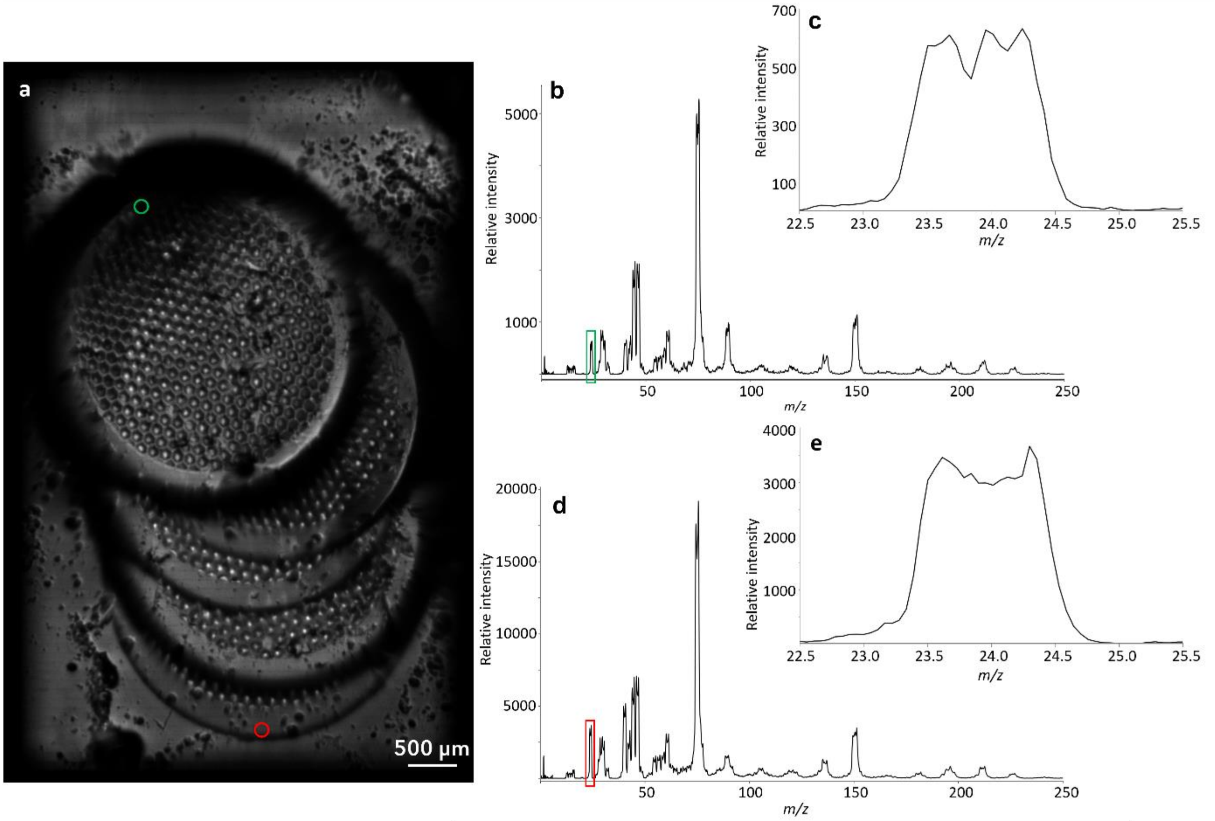

S4. (**a**) The selected regions on the top and bottom grids used to construct the mass spectra shown. The regions were chosen to be as close to the SEM point height measurements as possible. (**b**) The full mass spectrum obtained from the bent part of the top grid in the stack of grid mass image. Highlighted in green is the Na^+ peak, which is shown as an insert in (**c**). (**d**) The full mass spectrum obtained from the bottom grid in the stack of grid mass image. Highlighted in red is the Na^+ peak, which is shown as an insert in (**e**), which is slightly shifted by ~0.05 *m/z* towards larger m/z values compared to the Na^+ peak obtained in (**c**).

Figure S5:

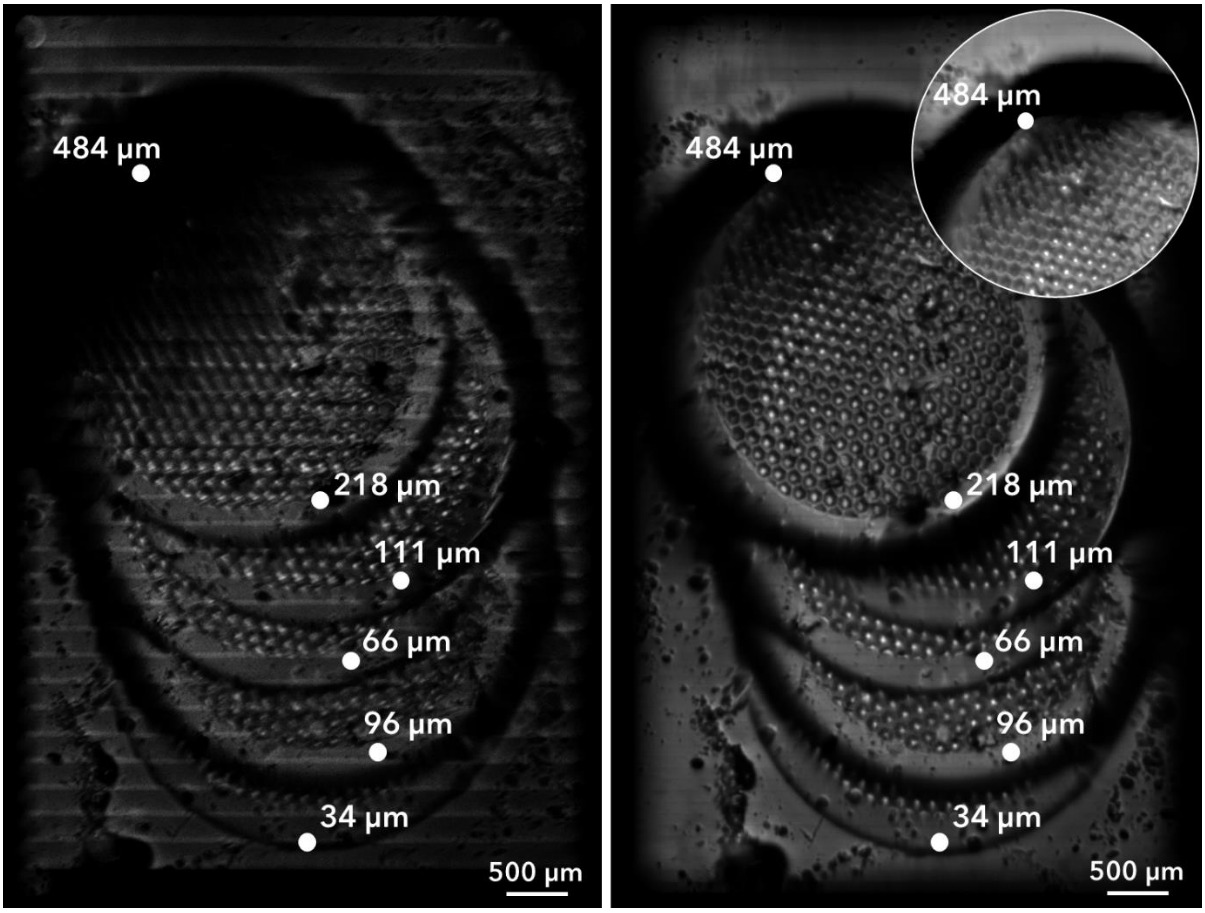

S5. Two FMM-TIC images of the stack of grids secured to an ITO slide with ink (also shown in S3, S4, and Figure 2). Point height measurements of each grid border were acquired relative to the ITO slide and are shown as white dots with the measured height. The left image was acquired with no manual C_{60} primary ion beam steering and with an unoptimized amount of row overlap. Even without manual beam steering, signal intensity and ion image focus are unchanged between 0 to 218 μm. Manually observing the location of the ion images and compensating for them by steering the C_{60} beam can improve image homogeneity and signal in uneven areas above 218 μm.

Figure S6:

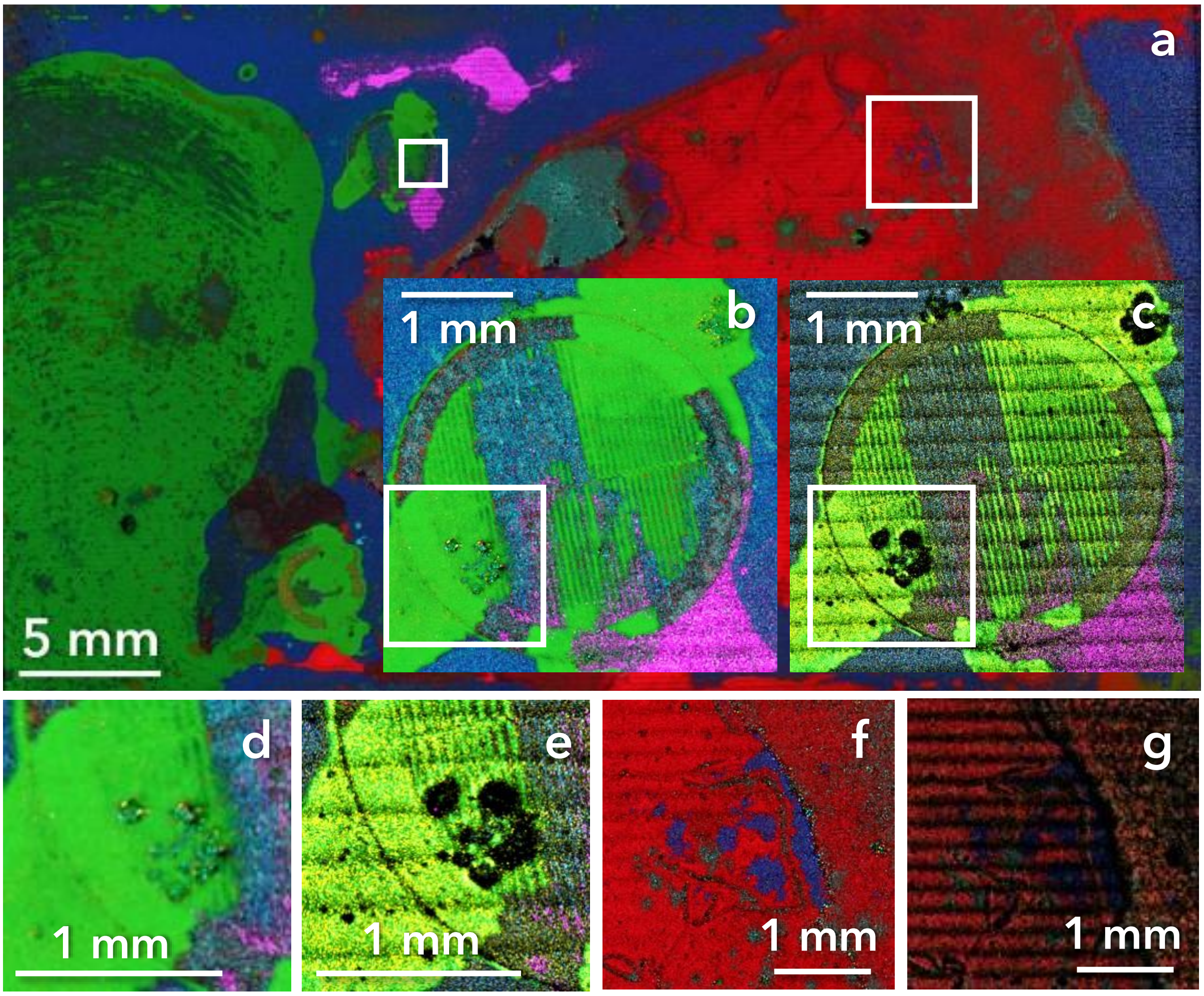

S6. (a) The multipass image observed in Figure 4b with a contrast-adjusted zoom-in of the top TEM grid (b) and corresponding zoomed-in area on the single-pass image (c). These zoom-ins show the high spatial resolution achieved and highlight the improvements in homogeneity and signal of the multipass image when compared to the singlepass (highlighted in the ability to observe signals in (d) that are absent or very dark in (e)). A zoom-ed in area of the lemon slice demonstrates the clear improvements in image homogeneity obtained in multipass-mode (f) when compared to singlepass-mode (g). Details such as the fine grid structure in (c and e) are somewhat less visible in (b and d) due to optimization and contrast adjustments performed on the multipass image (a, b, d, and f) in postprocessing that help to emphasize low-abundant signals these were not performed in the singlepass (c, e, and g) as they would result in extremely dark images.

Figure S7:

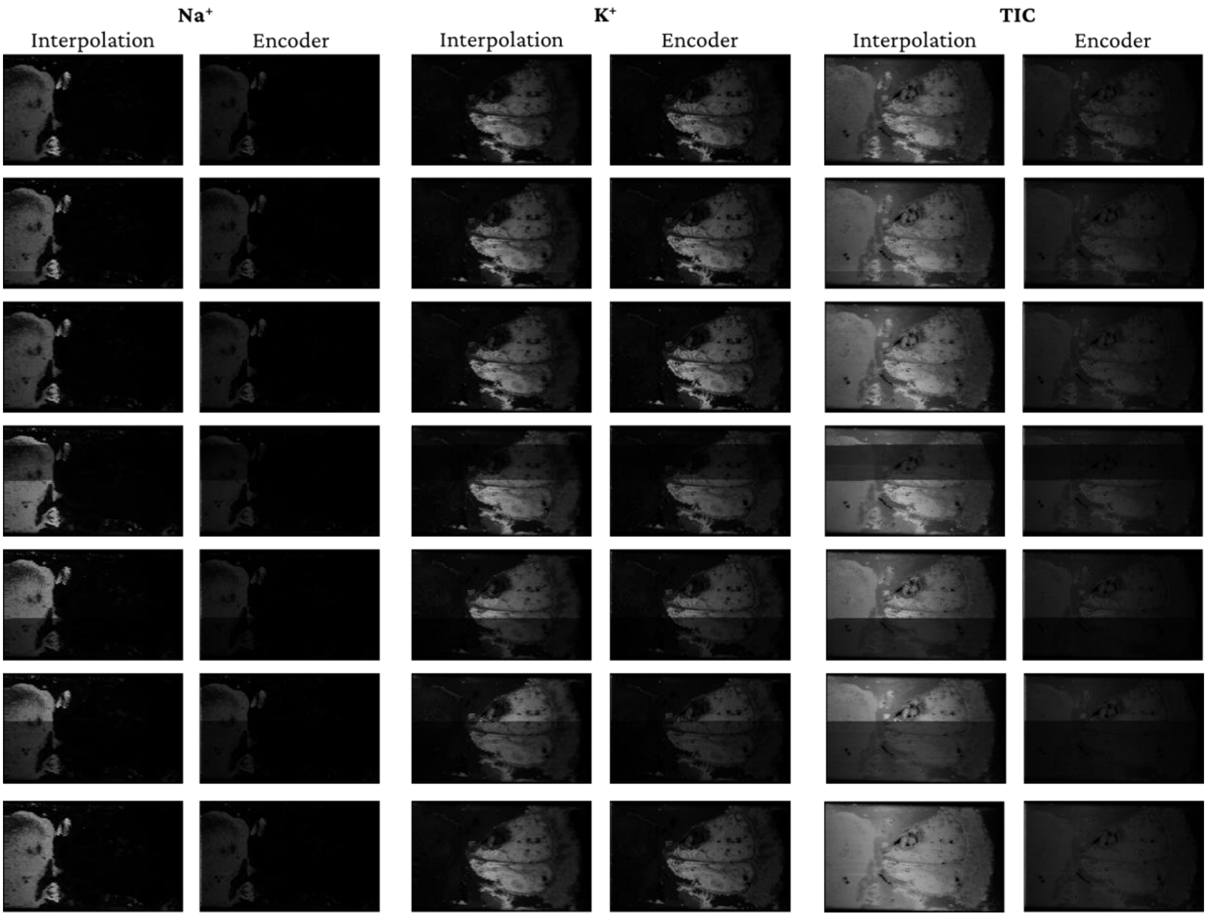

S7. Images of the seven replicate measurements showing the distributions of sodium (columns 1 and 2), potassium (columns 3 and 4), and total ion count (TIC) images (columns 5 and 6). Odd columns (1, 3, and 5) show images produced using the interpolated coordinates. Even columns (2, 4, and 6) show images produced using the encoder coordinates. The differences between encoder and interpolated coordinates observed for sodium and TIC images are due to visual intensity scaling. The difference in highest pixel intensity (and thus overall brightness differences) is explained by the encoder coordinates correctly assigning more time spent (and thus more ion counts) to the start and end borders of each row. This is due to the stage acceleration and deceleration at the start and end of the rows.

Figure S8:

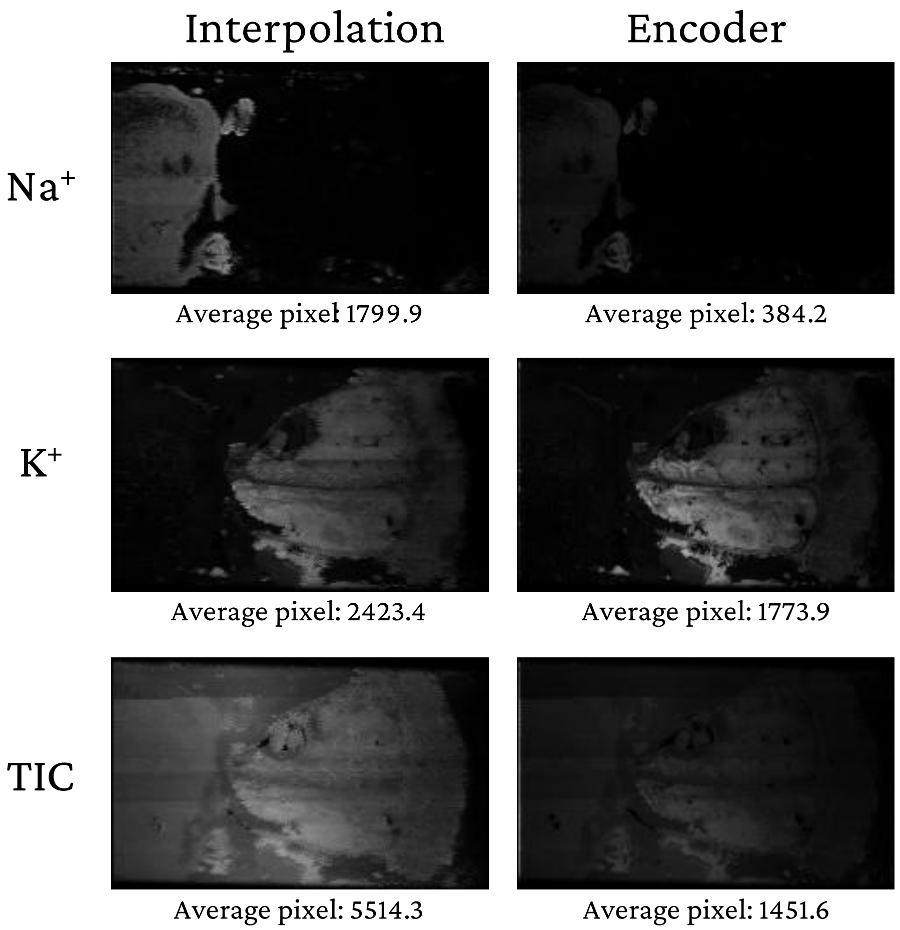

S8. Images of the standard deviation of the sodium (top row), potassium (middle row), and total ion count (TIC, bottom row) images shown in S6. Below each image is the value of the average pixel in the image. Due to intensity normalization the standard deviations of each image are relative to only that class of images. Although not necessarily indicative of image quality, the average pixel standard deviations of the interpolated coordinates (left column) are higher than those of images produced by the encoder coordinates (right column). Also, the lack of reproducibility of the interpolated coordinates can be observed in the artifact-laiden TIC image (bottom left).

Figure S9:

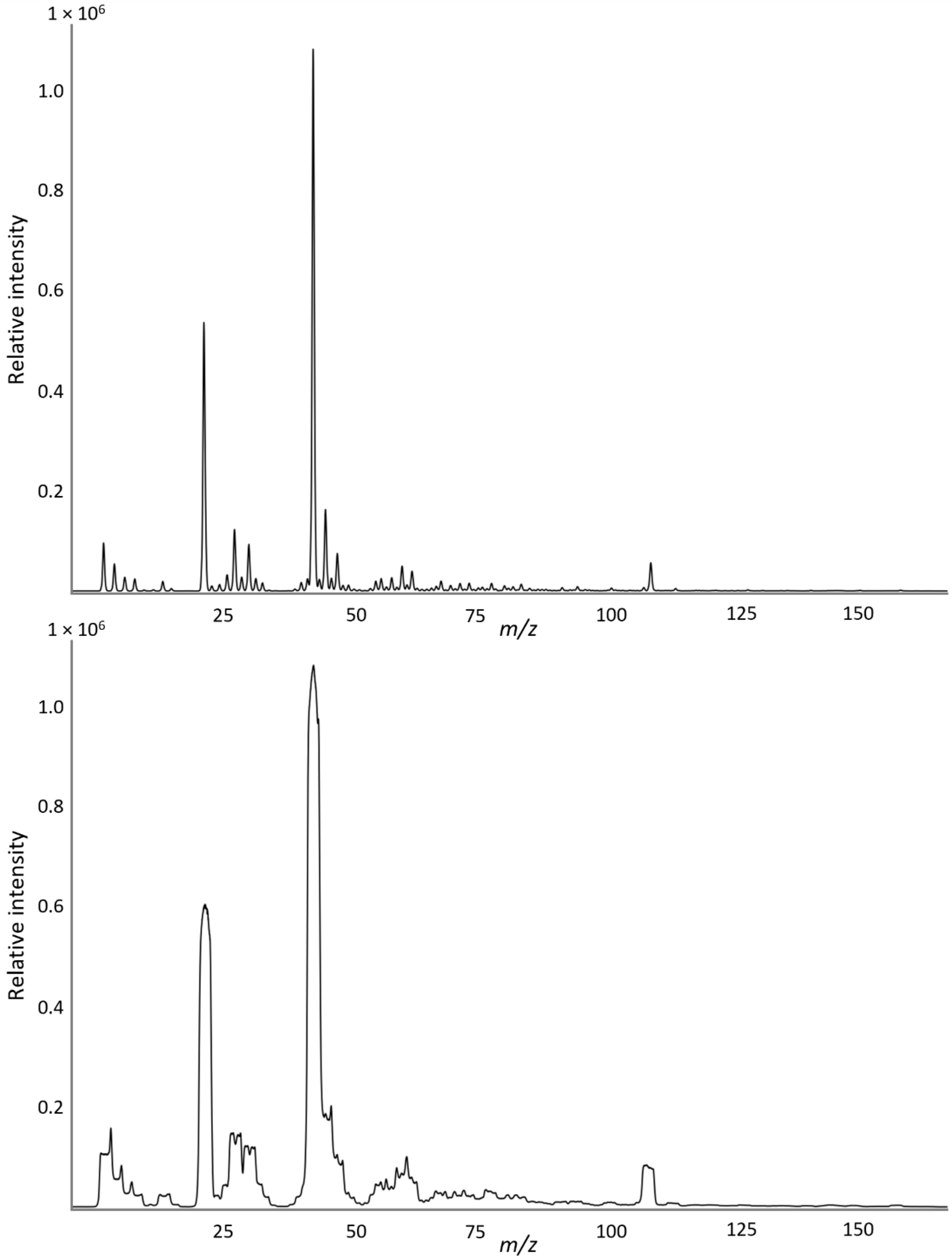

S9. Averaged mass spectrum of the composed surface taken with a primary ion pulse width of 150 ns (top) and 500 ns (bottom). The peak at *m/z* ~23 corresponding to Na^+ has a mass resolution ($m/\Delta m_{50\%}$) of ~75 in the top spectrum and a mass resolution of ~15 in the bottom spectrum.
